# Blood absorption capacity of different xenograft bone substitutes. An in-vitro study

**DOI:** 10.4317/jced.56317

**Published:** 2019-11-01

**Authors:** Octavi Ortiz-Puigpelat, Andreia Simões, Jordi Caballé-Serrano, Federico Hernández-Alfaro

**Affiliations:** 1 Assistant Professor Department of Oral and Maxillofacial Surgery. Universitat Internacional de Catalunya. Director of Clínica Dental Ortiz-Puigpelat. Barcelona, Spain; 2 DDS, MS. Resident Student of Department of Oral and Maxillofacial Surgery. Universitat Internacional de Catalunya; 3 DDS, MS, PhD. Assistant Professor Department of Oral and Maxillofacial Surgery. Universitat Internacional de Catalunya; 4 MD, DDS, PhD, FEBOMS. Professor & Chairman Department of Oral & Maxillofacial Surgery. Universitat Internacional de Catalunya. Director InstitutoMaxilofacial. Teknon Medical Center

## Abstract

**Background:**

Commercially available xenograft blocks, claim to have adequate characteristics to interact with biological media and thus permitting biological fluid absorption. The objective of this in vitro study was to compare the blood absorption capacity of four different xenograft block materials of different composition of collagen and porosity.

**Material and Methods:**

Four brands of xenograft block materials were used (NuOss®, Bio-Oss®, Osteobiol® and Smartbone®). Five samples of each brand were analyzed, making a total of 20 tests. Human blood was used as the absorption liquid for the present experiment. The time period, in which the block remains in contact with the blood, was registered at 30 seconds (T1), 60 seconds (T2) and 5 minutes (T3). The xenograft blocks were evaluated according to their absorption capacity.

**Results:**

The absorption capacity of the different biomaterials were statistical significant different (*p*<0,001) at T1, T2 and T3 time points. At 30 seconds, Smartbone® absorbed significantly less blood than NuOss® and Bio-Oss®, however, without differences comparing with Osteobiol®. The NuOss®, Bio-Oss® and Osteobiol® did not register any significant difference between them. At 60 seconds, the Smartbone® absorbed significantly less blood than the other biomaterials.

**Conclusions:**

The NuOss® was significantly superior than Osteobiol®, but without differences relatively with Bio-Oss®. Also the Bio-Oss® and Osteobiol® did not register any difference between them. At 5 minutes, the Smatbone® continued to significantly absorbed less blood than any other biomaterial, nevertheless, NuOss®, Bio-Oss® and Osteobiol® not register again any significant difference between them. Despite of small sample size, it can be concluded that NuOss® was superior, in terms of blood absorption capacity, comparing with the other block biomaterials at 30 seconds, 60 seconds and 5 minutes. However, more investigation in a clinical setting are needed to know the clinical implications of the absorption capacity of such biomaterials.

** Key words:**Blood absorption, osteoconduction, xenograft, bone regeneration.

## Introduction

The use of particulated xenografts in combination with resorbable membranes is the most well-documented technique for the regeneration of localized alveolar bone defects (1-3). Moreover, the survival of implants placed in regenerated bone is comparable to those placed in native bone (4,5). However, when particulated grafts are used, mechanical stability can be compromised (6). In such situations, the use of either non-resorbable devices or autogenous bone blocks are recommended (7,8). On the other hand, some clinical disadvantages such as the need of a donor surgical site and removal of the non-resorbable device are associated with these techniques (9,10). Recent investigations are showing promising results, both in clinical and preclinical settings with the use of bone substitutes materials in block format (11-19). However, some preclinical studies, reported connective tissue infiltration resulting in low levels of new bone formation. Such infiltration was located at the outer surface of the blocks away from the recipient site. Furthermore, the percentage of new bone formation was scarce and limited at the bottom part of the blocks and at the interface between the recipient bone and the block graft (14,18,19). Other clinical investigations with the use of interpositioned xenograft blocks, showed more favorable results in terms of volume stability and percentage of new bone formation (15,20). Scarano *et al.* reported that most of the graft particles that were interpositioned in inlay regeneration, were infiltrated by biological fluids and filled with newly formed bone, achieving a percentage of new bone formation of 44% (20). Therefore, in vivo osteoconduction property of xenograft biomaterials may be influenced by an adequate structural characterization of bone substitute biomaterials that enhance the interaction between the biomaterial and biological media that ultimately favors the absorption of biological fluids and cell penetration (21-24).

Wettability is the ability of any solid surface to be wetted when in contact with a liquid, and therefore, is an important characterization of biomaterials that can influence their absorption capacity of biological fluids (24).

Commercially available xenograft blocks, claim to have adequate characteristics to interact with biological media and thus permitting biological fluid absorption. Therefore, the objective of the present pilot study was to quantify the absorption capacity of some commercially available xenograft bone blocks.

## Material and Methods

This study was performed in the laboratory of Facultat d’Odontologia of Universitat Internacional de Catalunya.

Due to the fact that there are no previous studies about this subject, a pilot study with small sample size was designed.

**Figure 1:**
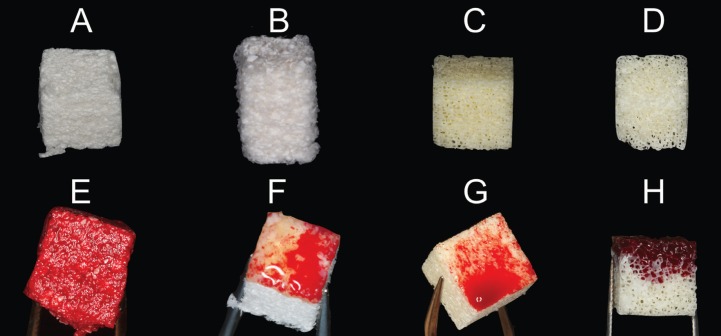
From A to D, block specimens before starting the study. A, bovine-80%; B, bovine-63%; C, bovine-27%; and D, equine-33%. From E to H, block specimens after the experiment.

Only xenograft blocks were analyzed to reduce bias. The following brands were evaluated: NuOss® (ACE Surgical Supply Company Inc., USA), Bio-Oss® (Geistlich Pharma AG, Switzerland), OsteoBiol® (Tecnoss, Italy) and Smartbone® (Ibi SA, Switzerland) (Fig. 1). Five samples of each material brand were analyzed, making a total of 20 observations. Each brand presented different commercially available block sizes. The block size of each block was selected in order to be similar to each other (NuOss® 8X9X9 mm, Bio-Oss® 0.2-0.3 cm3, OsteoBiol® 10X10X10 mm and Smartbone® 10X10X10 mm). Most of them were bovine origin except for OsteoBiol® which was equine origin. Smartbone® block graft was composed by bovine particles mixed with resorbable polymers. The blocks presented with different percentage of collagen fragments: NuOss® 5%, Bio-Oss® 10%, OsteoBiol® 35% and Smartbone® some particles. The total porosity of each block was also different between brands: NuOss® 80%, Bio-Oss® 63.5%, OsteoBiol® 33.1% and Smartbone® 27% .A specific name for each brand was designated related to its characteristics: bovine-80% (NuOss®), bovine-63% (Bio-Oss®), equine-33% (OsteoBiol®) and bovine-27% (Smartbone®).

The absorbent liquid used in the present study was human blood. The blood was collected using tubes with an anticoagulant solution of potassium citrate in order to avoid coagulation.

**Figure 2:**
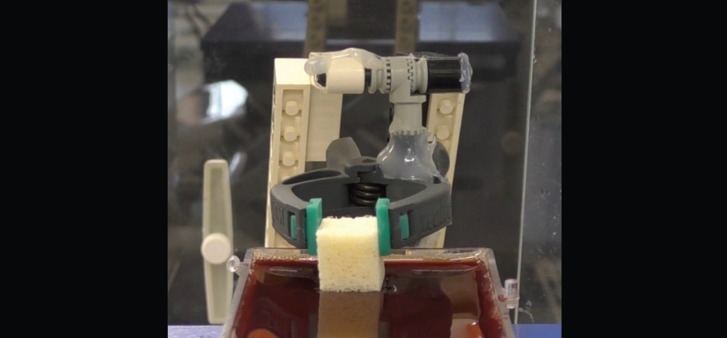
Light weight device holding the biomaterial and placed on top of the precision scale.

The method of measure the differences in weight was using a precision scale (Sartorius Extend Analytical Balances) with the precision of 0.0001 grams. In order to minimize the noise in the balance and making it very sensitive to changes in weight, a method of weighting described in the literature was used (21). It consisted in fixing the sample in a light weight structure located on the precision scale (Fig. 2), then the blood was brought into contact by a movable stage outside the precision scale. Previously, the lightweight structure was weighted and tared to 0 value. Then, the sample was fixed at the lightweight structure in order to measure the initial weight of the blocks, before starting the experiment. When blood contacted the sample, the movable stage was stopped. With this method, the increases in weight could be observed and registered and since the sample was held in a constant position there were negligible buoyancy effects (18). As the experiment continues, the samples increased their weight as more liquid was drawn into the pores of the material.

The time period, in which the block remained in contact with the blood, was measured with a stopwatch, after 30 seconds (T1), 60 seconds (T2) and 5 minutes (T3). The weight was recorded in each time interval. This process was repeated with the different blocks that were analyzed.

-Statistical analysis

Due to the exploratory nature of this study, no sample size calculation was performed. Statistical analyses were performed using a nonparametric model of Brunner-Langer and post-hoc comparisons with Bonferroni corrections. A SPSS15.0® and R.3.0.2® programs were used, with a significance level of 5% (α=0.05).

## Results

General results of the different weights after the observation time points are shown in  Table 1. In Figure 1, are shown the aspect of the blocks after the experiment. In  Table 2 are shown the mean gain the different groups between T0 and the different time points (T1, T2 and T3). Also, in order to help visualize the changes between groups and the different time points, the initial weights were normalized to 1 value ( Table 3).

**Table 1:**
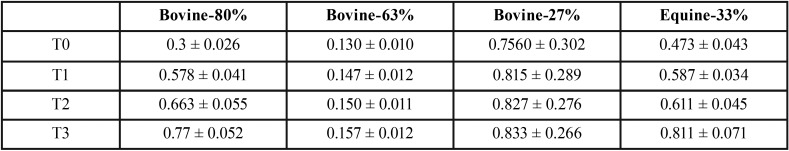
Mean values and standard deviation of the different weights obtained at the different time points. Values expressed in miligrams (mg).

**Table 2:**
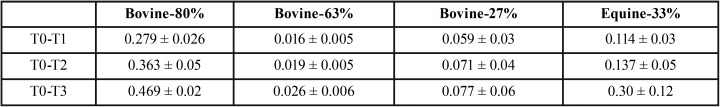
Differences of weights between T0 and the different time points: T1, T2 and T3. Values expressed in mg.

**Table 3:**
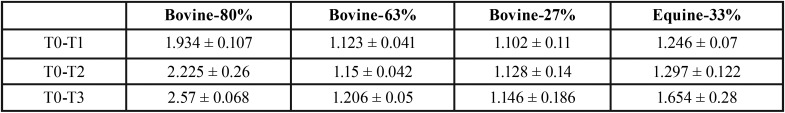
Differences of weights of the different groups at different time points with normalized initial weight to 1. Values expressed in mg.

**Figure 3:**
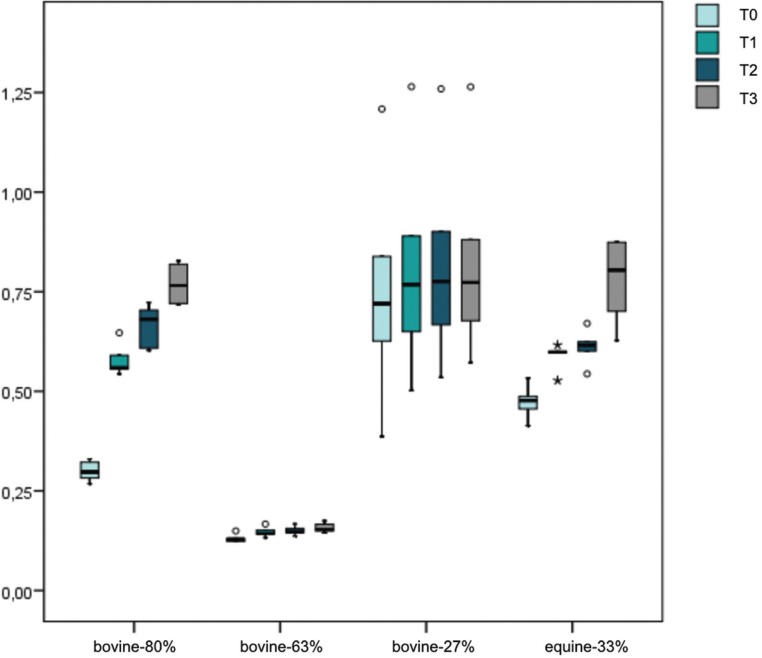
Box plot graph with the distribution of the weights of the different biomaterials at different time intervals.

A box plot graph was elaborated to exhibit the distribution of the weight values between groups and time observations (Fig. 3). It can be observed in such graph that the bovine-63% and bovine-27% groups present a limited absorption capacity, since the weight values remain stable throughout the observation times. However, it is only a visual impression, due to the lighter initial weights of the bovine-63%.

The global changes of the biomaterials were also analyzed. Such analysis was divided into three different sections corresponding to the three time points evaluated at T1, T2 and T3.

At 30 seconds (T0-T1)

Between T0 and T1, the blocks changed significantly their weight, meaning that there is a time effect (*p*<0.001). However, the gain of weight is not homogeneous between the groups. An interaction effect exists (*p*<0.001). In  Table 4 are shown the comparisons of the weight change between the period T0-T1. It can be noticed that the bovine-80% and bovine-63% absorbed significantly more blood than bovine-27%. No significant differences were observed between bovine-80% and bovine-63% (*p*=0.239). The equine-33% did not registered any significant differences when compared with all of the other biomaterials.

**Table 4:**
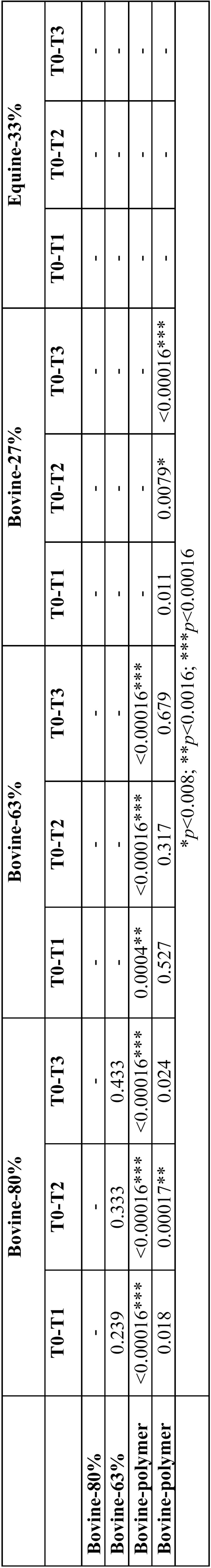
Results of Brunner-Langer model with Bonferroni adjustment in order to compare the changes in weight between pair of groups from T0-T1, T0-T2 and T0-T3.

At 60 seconds (T0-T2)

Between T0 and T2, the blocks change significantly their weight, showing again the time effect (*p*<0.001). However, the magnitude of this alteration was different according with the type of biomaterial (*p*<0.001). The bovine-27% absorbed significantly less blood than the others. The bovine-80% blocks were significantly superior to equine-33% (*p*=0.00017). The equine-33% was significant superior to bovine-27% but without differences when compared with bovine-63% ( Table 4).

At 5 minutes (T0-T3)

Between T0 and T3, the blocks change significantly their weight (*p*<0.001). However, the magnitude of this alteration was different according with the type of biomaterial (*p*<0.001). The bovine-27% absorbed significantly less blood than the others. The bovine-80%, the bovine-63% and the equine-33% did not present any significant difference between them ( Table 4). Also, despite of the significant differences between equine-33% and bovine-80% at T2, favoring the later, they did not register any significant differences at T3 due to the big absorption capacity of the equine-33% between T2 and T3. These differences became more equal after 5 minutes, although more favorable results for the bovine-80% group (*p*=0.024).

Global study model (T0, T1, T2 and T3)

The blocks increased significantly their weights throughout the entire period of the experiment (*p*<0.001). However, with a different pattern depending on the biomaterial (*p*<0.001). The change of weight between T0 and T1 were weaker in bovine-63% and bovine-27% groups when comparing with bovine-80% and equine-33%. The bovine-80%, despite started the initial weight lower than equine-33%, at T1, their weights had become equal and even at T2, bovine-80% was higher than equine-33%. Finally, both weights at T3 were proportional to T0.

Although mechanical stability of the blocks was not part of the objective of the present study, the bovine-80% blocks changed their shape after the experiment, showing a lack of mechanical stability. However, all of the other blocks did not change the form and remained stable from the beginning.

## Discussion

Up to the author’s knowledge, no similar studies were published in the literature, therefore comparisons with other studies could not be made.

All the blocks used in the present study are commercially available and were not manufactured for the study.

One of the limitations of the study was the small sample size. However, this pilot study showed interesting results in regard of the blood absorption of the different xenograft blocks. Generally, all blocks augment their weight during the experiment (*p*<0.001). However, this increment in weight was different between the test groups (*p*<0.001). At T1, bovine-80% and bovine-63% are clearly superior than bovine-27%. Meanwhile, equine-33% tends to have the same absorption capacity than bovine-80% and bovine-63%, but with less magnitude. These differences observed at a very short period of time, can indicate a superior advantage of bovine-80% and bovine-63% over the other biomaterials since early blood-mediated inflammatory response triggers the cascade of initial bone repair (25,26). Results of the weight gain at T2 and T3 of the different test groups reveled that bovine-27% was the block with less absorption capacity. The absorption capacity is more progressive and linear for bovine-80% while the equine-33% highlights a noticeable weight gain between T2 and T3. Equine-33% group tends to a more important absorption capacity than bovine-63% group. Bovine-80%, revealed to be the most effective biomaterial for the blood absorption capacity, since it is significantly better than bovine-27% at any time and globally exhibiting a strong tendency to overcome bovine-63% and equine-33%.

These differences in blood absorption capacity, may affect the percentage of new bone formation during guided bone regeneration (22,24). In clinical studies, this formation in block bone substitute grafts, basically is found at the interface between the block and the recipient site and usually tends to be around 5.9% (19). However, when the xenografts blocks are interpositioned, where two bone walls are present, the percentage of new vital bone tends to rise up till 44%, without gaps nor connective tissue between the biomaterial and bone interface (20). These findings, reinforce the idea that, enhanced blood absorption capacity of blocks will make them less dependent on the amount of blood supply sources of the defect sites.

Wettability is not the only characteristic that can influence blood absorption capacity of biomaterials, also, micro and macro-porosity, can influence as well (27). Although the present study was not designed to relate the blood absorption capacity with such factors, it could be observed a proportional relation between them, since the groups with higher percentage of total porosity obtained higher values of blood absorption. This can have an important clinical implication because it is known that increased porosity facilitate bone ingrowth (23). On the other side, as reported in the literature, if total porosity is increased it may affect the mechanical strength of the biomaterial (23). Despite not being the objective of the study, we could corroborate that bovine-80% and bovine-63% blocks with higher percentages of total porosity, their shape and integrity were slightly deformed at T3. Therefore, the use of these blocks could not be used in non-contained defects, but instead, it would be more reasonable to use them in contained defects such as socket preservation.

Within the limitations of this pilot study it can be concluded that the blood absorption of the different groups increased over time. However, with a different magnitude depending on the biomaterial. Bovine-83% group appears to be the most effective, followed by equine-33% and bovine-63%. The latter two groups having no significant differences between them. Bovine-27% exhibit the poorest results in terms of blood absorption. More *in-vitro* and *in-vivo* studies are required to ascertain the clinical implications of absorption capacity of bone substitute grafts in alveolar bone regeneration.
